# BHBA Suppresses LPS-Induced Inflammation in BV-2 Cells by Inhibiting NF-****κ****B Activation

**DOI:** 10.1155/2014/983401

**Published:** 2014-04-06

**Authors:** Shou-Peng Fu, Su-Nan Li, Jian-Fa Wang, Yang Li, Shan-Shan Xie, Wen-Jing Xue, Hong-Mei Liu, Bing-Xu Huang, Qing-Kang Lv, Lian-Cheng Lei, Guo-Wen Liu, Wei Wang, Ju-Xiong Liu

**Affiliations:** ^1^College of Veterinary Medicine, Jilin University, Changchun 130062, China; ^2^College of Animal Science and Veterinary Medicine, Heilongjiang Bayi Agricultural University, Daqing 163319, China

## Abstract

**β**-Hydroxybutyric acid (BHBA) has neuroprotective effects, but the underlying molecular mechanisms are unclear. Microglial activation plays an important role in neurodegenerative diseases by producing several proinflammatory enzymes and proinflammatory cytokines. The current study investigates the potential mechanisms whereby BHBA affects the expression of potentially proinflammatory proteins by cultured murine microglial BV-2 cells stimulated with lipopolysaccharide (LPS). The results showed that BHBA significantly reduced LPS-induced protein and mRNA expression levels of iNOS, COX-2, TNF-**α**, IL-1**β**, and IL-6. Blocking of GPR109A by PTX resulted in a loss of this anti-inflammatory effect in BV-2 cells. Western blot analysis showed that BHBA reduced LPS-induced degradation of I**κ**B-**α** and translocation of NF-**κ**B, while no effect was observed on MAPKs phosphorylation. All results imply that BHBA significantly reduces levels of proinflammatory enzymes and proinflammatory cytokines by inhibition of the NF-**κ**B signaling pathway but not MAPKs pathways, and GPR109A is essential to this function. Overall, these data suggest that BHBA has a potential as neuroprotective drug candidate in neurodegenerative diseases.

## 1. Introduction

In the healthy central nervous system (CNS), microglial cells are the immune cells belonging to the innate part of the immune system. Beneficial functions of microglia include release of trophic and anti-inflammatory factors [1, 2] and clearance of cellular debris [3]. In contrast, microglial cells can exhibit detrimental capacities involving overproduction of neurotoxic factors like nitric oxide, superoxide radicals, and TNF-*α* [4, 5]. Activation of microglia has been implicated in the pathogenesis of a variety of neurodegenerative diseases, including multiple sclerosis, Parkinson's disease, Huntington's disease, and Alzheimer's disease [6–8]. Activation of microglia and consequent release of proinflammatory and/or cytotoxic factors such as TNF-*α*, IL-1*β*, IL-6, nitric oxide, reactive oxygen species, inducible nitric oxide synthase (iNOS), and cyclooxygenase-2 (COX-2) are believed to contribute to neurodegenerative processes [9, 10]. LPS is a bacterial endotoxin used to study experimentally induced activation of microglia [11]. Mechanistically, LPS stimulates Toll-like receptor 4 (TLR4) to activate nuclear factor-*κ*B (NF-*κ*B) and mitogen-activated protein kinases (MAPKs) family [12], which are classified into at least three components: extracellular signal-regulated kinases (ERKs), c-Jun N-terminal kinase (JNK), and p38 MAPK, which have been implicated in the release of immune-related cytotoxic factors such as iNOS, COX-2, and proinflammatory cytokines (TNF-*α*, IL-1*β*, and IL-6) [11, 13].

As an important intermediate of amino and fatty acid catabolism, BHBA like glucose can be used by the brain to provide energy particularly for suckling newborns [14]. The impact of BHBA on neuroprotection has recently been summarized [15]. However, the underlying molecular mechanisms are still not resolved. GPR109A (PUMA-G in mice and HM74A in humans) is a seven-transmembrane G-protein-coupled receptor of the Gi family that is expressed mainly in white adipocytes and immune cells such as monocytes and neutrophils [16]. BHBA has been identified as an endogenous ligand of GPR109A [17]. Zandi-Nejad et al. have proved that HCA2 (GPR109A) suppresses macrophage proinflammatory function by inhibiting proinflammatory cytokine production, LDL uptake, and chemotaxis [18]. Digby et al. have found that nicotinic acid has anti-inflammatory action in human monocytes via GPR109A [19]. In this study we have proved BHBA receptor GPR109A is expressed in murine microglial BV-2 cells. So, we hypothesized that BHBA has the potential to act directly on microglia to inhibit proinflammatory proteins that may contribute to its neuroprotective effects in vivo.

In the present study, we attempted to elucidate the anti-inflammatory potential of BHBA on the inflammatory response induced by LPS in murine microglial BV-2 cells. To further investigate the underlying mechanisms, the involvement of I*κ*B-*α*, NF-*κ*B, and MAPKs was also examined. The present study provides information revealing BHBA as a potential candidate compound with anti-inflammatory actions and suggests a scientific basis for further investigation of BHBA against neuroinflammatory conditions.

## 2. Methods

### 2.1. Cells and Treatments

The immortalized mouse microglial cell line BV-2 cells were seeded on 6 cm tissue culture plates and maintained in DMEM (Hyclone, Logan, UT, USA) supplemented with 10% (v/v) FCS (Hyclone, Logan, UT, USA) and 1% (v/v) penicillin/streptomycin solution (Hyclone, Logan, UT, USA) at 37°C in 5% CO_2_ atmosphere. The culture medium was changed twice a week and cultures were passaged at 80% confluence after trypsinisation (0.05%, w/v). Changes in cell morphology and growing conditions were carefully monitored using an inverted microscope. To reduce mitogenic effects, BV-2 cells were precultured in serum-free DMEM for at least 4 h. Six groups of BV-2 cells were subjected to various treatments. In group 1, the cells were incubated in serum-free DMEM. In group 2, the cells were treated with 1.5 mM BHBA (Sigma, St. Louis, MO, USA). In group 3, the cells were treated with 1 *μ*g/mL LPS (Sigma, St. Louis, MO, USA). In groups 4, 5, and 6, the cells were treated with 0.5 mM, 1.0 mM, and 1.5 mM BHBA for 1 h and then stimulated with LPS (1 *μ*g/mL). Cell incubations were for 5 min–24 h, as indicated in the text.

### 2.2. RNA Extraction, Reverse Transcriptase PCR, and Quantitative Real-Time PCR Analysis

Total RNA was extracted from the cells using Trizol (Invitrogen, Carlsbad, CA, USA), according to the supplier's protocol. Total RNA was then treated with RNase-free Dnase I, subsequently quantified by measuring the absorbance at 260 and 280 nm and stored at −80°C until analysis. The extracted RNA was subjected to a RT-PCR using PrimeScript RT reagent Kit With gDNA Eraser (Takara Shuzo Co., Ltd., Kyoto, Japan). The mRNA levels of various genes were evaluated by quantitative polymerase chain reaction (qRT-PCR) analysis using the SYBR Green QuantiTect RT-PCR Kit (Roche, South San Francisco, CA, USA), performed in triplicate for each sample. The relative expression levels for iNOS, COX-2, TNF-*α*, IL-1*β*, IL-6, and GPR109A were calculated relative to *β*-actin (the normalizer) using the comparative cycle threshold method. The primer sequences for the tested genes are shown in Table 1.

### 2.3. ELISA

BV-2 cells seeded in 24-well plates were pretreated with various concentrations of BHBA for 1 h followed by stimulation with LPS (1 *μ*g/mL) for another 24 h. After stimulation, culture media were collected and centrifuged at 13000 rpm for 3 min. The amounts of cytokines in the supernatants for TNF-*α*, IL-1*β*, and IL-6 were determined by ELISA (BioLegend, San Diego, CA, USA) according to the manufacturer's instructions. Three replicates were carried out for each of the different treatments.

### 2.4. Western Blot Analysis

Cells were harvested with ice-cold PBS and centrifuged at 14000 ×g for 3 min at 4°C. Nuclear and cytosolic extracts were prepared using a Nuclear and Cytoplasmic Protein Extraction Kit (Beyotime Institute of Biotechnology, Jiangsu, China) according to the manufacturer's instructions. Concentration of the protein was measured using a bicinchoninic acid protein assay kit (Beyotime Co., China). A total of 50 *μ*g of protein was resolved onto 10% SDS-polyacrylamide gel electrophoresis (SDS-PAGE) and transferred onto immunoblot polyvinylidene difluoride membranes (Chemicon International, Millipore, Billerica, MA, USA). The blots were blocked with 5% nonfat milk in Tris-buffered saline with 0.1% Tween (TBS-T) for 1 h, washed three times with TBS-T, and incubated overnight at 4°C with primary antibodies iNOS (1 : 2000), COX-2 (1 : 1000) (Abcam, Cambridge, CA, USA), phospho-ERK1/2 (1 : 2000), ERK1/2 (1 : 2000), phospho-p38 (1 : 2000), p38 (1 : 1000), phospho-JNK (1 : 1000), JNK (1 : 2000), I*κ*B-*α* (1 : 1000) (Cell Signaling Technology, Danvers, MA, USA), NF-*κ*B/RelA (1 : 1000), GPR109A (1 : 300), PCNA (the marker of nuclear fraction) (1 : 1000), and *β*-actin (the marker of cytoplasm fraction) (1 : 2000) (Santa Cruz Biotechnology). Blots were then washed four times for 15 min each in TBS-T and incubated with horseradish peroxidase-labeled secondary goat anti-rabbit (1 : 2000; Santa Cruz Biotechnology) or rabbit anti-goat (1 : 2000; Santa Cruz Biotechnology) for 1 h at room temperature. Blots were again washed four times for 15 min each in TBS-T. Membranes were visualized with enhanced chemiluminescence (Pplygen Co., China).

### 2.5. Statistical Analyses

Results were expressed as means ± SD. Data were analyzed by using statistical software package SPSS 12.0 (SPSS Inc., Chicago, IL, USA). Groups were compared by one-way analysis of variance (ANOVA) followed by the least significant difference test. A *P* value of less than 0.05 was considered statistically significant, and values less than 0.01 were considered markedly significant.

## 3. Results

### 3.1. Expression of GPR109A in Murine Microglial BV-2 Cells

**Figure 1:**
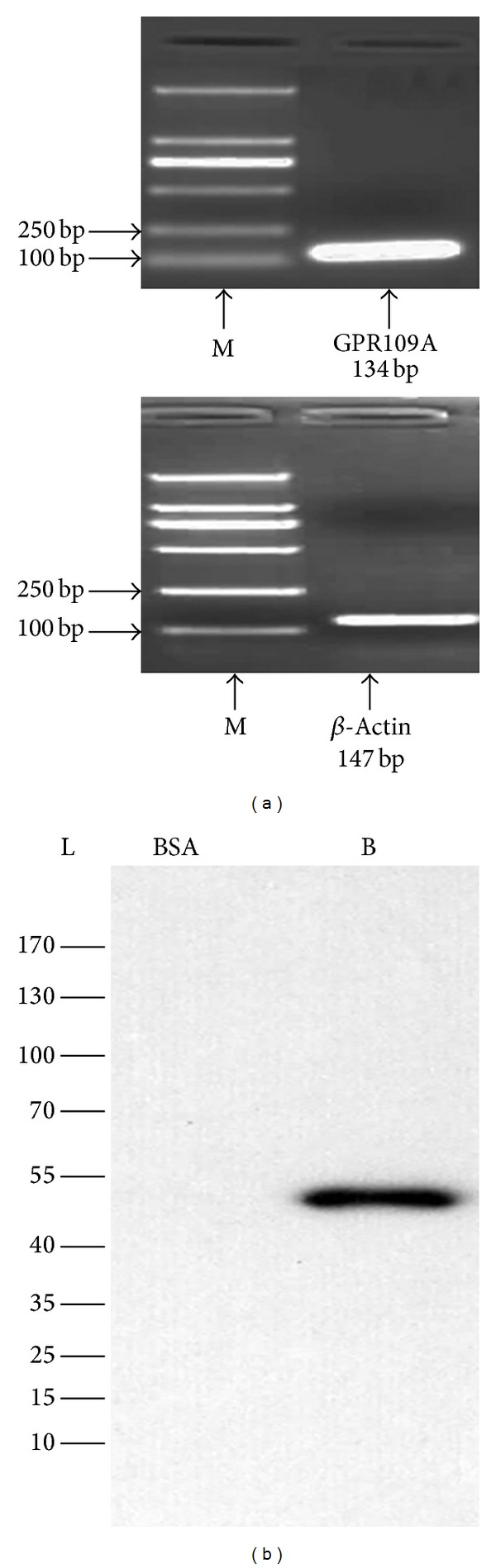
Expression of GPR109A in BV-2 cells. (a) RT mixtures from BV-2 cells were carried out to detect GPR109A mRNA expression by PCR amplification (M, 2000 bp DNA marker). PCR products were visualized by 2% agarose gel electrophoresis; the expected 134 bp GPR109A was detected in BV-2 cells. (b) Western blot of GPR109A in BV-2 cells showing a specific band of the expected size at approximately 50 kDa (lanes: L: protein ladder; BSA: bovine serum albumin; B: BV-2 cells).

GPR109A is the functional receptor of BHBA, and its mRNA (Figure 1(a)) and protein (Figure 1(b)) were detected in BV-2 cells. So, BHBA has the potential to act on BV-2 cells via GPR109A.

### 3.2. BHBA Inhibits LPS-Induced Protein and mRNA Expression of iNOS and COX-2 in BV-2 Cell

**Figure 2:**
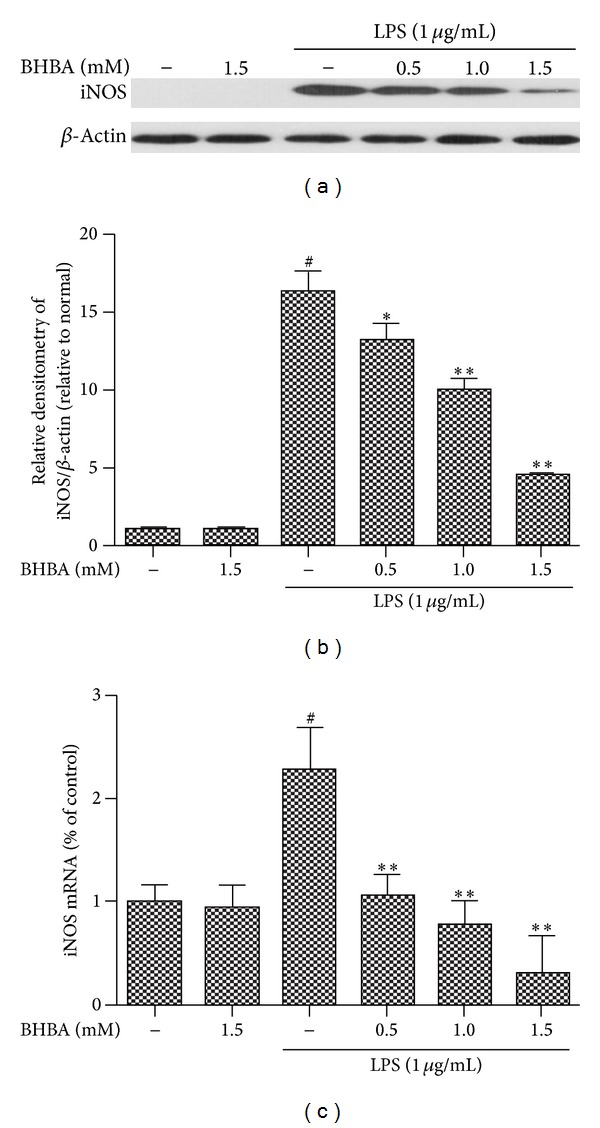
Effects of BHBA on LPS-induced protein and mRNA expression of iNOS in BV-2 cells. Cells were pretreated with BHBA (0.5, 1.0, and 1.5 mM) 1 h prior to incubation of LPS (1 *μ*g/mL) for 4 h. The levels of protein and the corresponding mRNA were determined by western blotting and quantitative real-time PCR as described in Section 2. (a) and (b) show protein expression of iNOS and (c) shows the corresponding mRNA data. The relative mRNA level was normalized to *β*-actin mRNA; the relative protein levels were quantified by scanning densitometry and normalized to *β*-actin. Results are expressed as mean ± SD for each group from three independent experiments. ^#^Significant compared with control alone, *P* < 0.05. *P < 0.05 and **P < 0.01 versus the BHBA-untreated LPS-stimulated group.

**Figure 3:**
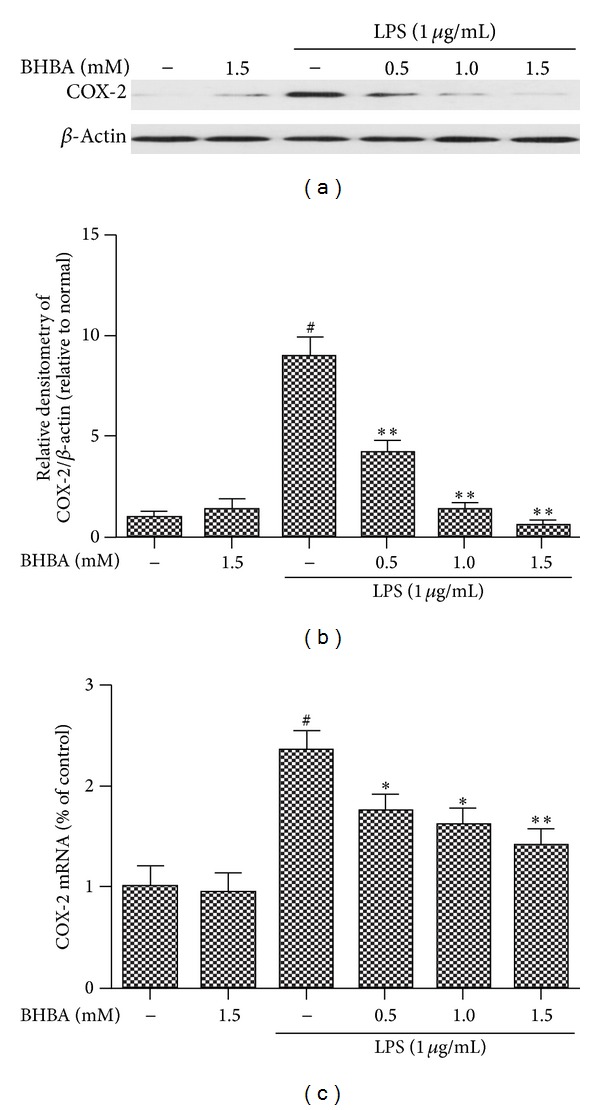
Effects of BHBA on LPS-induced protein and mRNA expression of COX-2 in BV-2 cells. Cells were pretreated with BHBA (0.5, 1.0, and 1.5 mM) 1 h prior to incubation of LPS (1 *μ*g/mL) for 4 h. The levels of protein and the corresponding mRNA were determined by western blotting and quantitative real-time PCR as described in Section 2. (a) and (b) show protein expression of COX-2 and (c) shows the corresponding mRNA data. The relative mRNA level was normalized to *β*-actin mRNA; the relative protein levels were quantified by scanning densitometry and normalized to *β*-actin. Results are expressed as mean ± SD for each group from three independent experiments. ^#^Significantly different when compared with control alone, *P* < 0.05. *P < 0.05 and **P < 0.01 versus the BHBA-untreated LPS-stimulated group.

iNOS and COX-2 are two important proinflammatory proteins correlated with LPS stimulation in microglia [11]. To investigate the effect of BHBA on LPS-stimulated microglial activation, BV-2 cells were pretreated with BHBA (0.5, 1.0, and 1.5 mM) for 1 h and then stimulated with LPS (1 *μ*g/mL) for 4 h. iNOS and COX-2 were examined by western blotting and quantitative real-time PCR assay. BHBA notably inhibited dose-dependently the increased protein and mRNA expression of iNOS (Figures 2(a)–2(c)) and COX-2 (Figures 3(a)–3(c)) stimulated by LPS.

### 3.3. BHBA Attenuates Expression of Proinflammatory Cytokines in LPS-Stimulated BV-2 Cells

**Figure 4:**
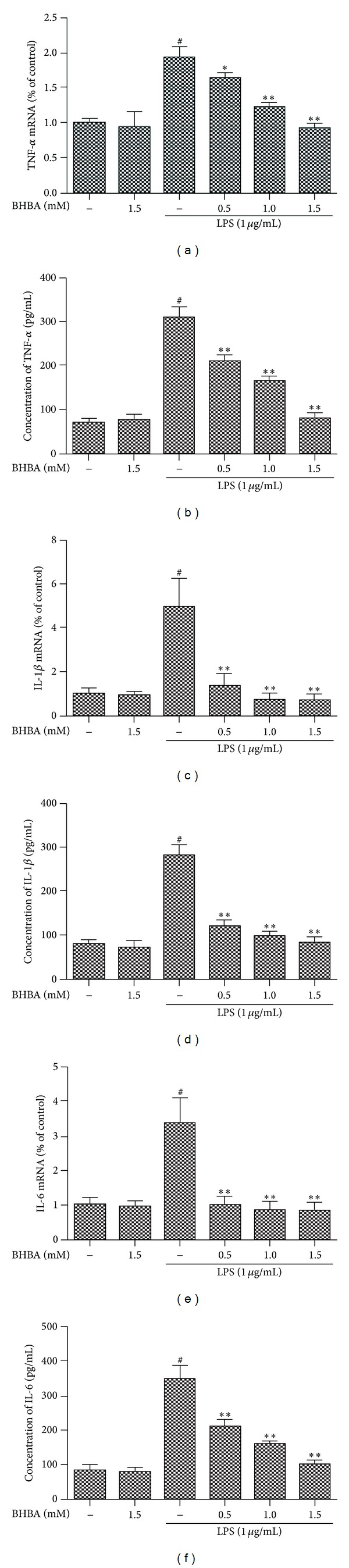
Effects of BHBA on LPS-induced the gene expression and protein secretion of proinflammatory cytokines (TNF-*α*, IL-1*β*, and IL-6) in BV-2 cells. Cells were pretreated with BHBA (0.5, 1.0, and 1.5 mM) 1 h prior to incubation of LPS (1 *μ*g/mL) for 4 h (mRNA) or 24 h (protein). Proteins and mRNA of TNF-*α* ((a), (b)), IL-1*β* ((c), (d)), and IL-6 ((e), (f)) were determined by ELISA and quantitative real-time PCR as described in Section 2. The relative mRNA level was normalized to *β*-actin mRNA. Results are expressed as mean ± SD for each group from three independent experiments. ^#^Significantly different when compared with control alone, *P* < 0.05. *P < 0.05 and **P < 0.01 versus the BHBA-untreated LPS-stimulated group.

Proinflammatory cytokines (including TNF-*α*, IL-1*β*, and IL-6) play important roles in inflammatory process. In order to investigate whether BHBA represses the production of these proinflammatory cytokines, BV-2 cells were stimulated with LPS (1 *μ*g/mL) in the presence or absence of BHBA (0.5, 1.0, and 1.5 mM). As shown in Figure 4, the significant increase of gene expression and protein secretion of TNF-*α* (Figures 4(a) and 4(b)), IL-1*β* (Figures 4(c) and 4(d)), and IL-6 (Figures 4(e) and 4(f)) resulting from the LPS stimulation was inhibited by BHBA in a dose-dependent manner in BV-2 cells. In order to determine the cytotoxicity of BHBA, we investigated the dose effect of BHBA on cell viability by MTT assay. BV-2 cells were incubated with various doses of BHBA for 24 h. The results of MTT assay showed that BHBA, even at a high concentration of 10 mM, did not affect cell viability (data not shown), demonstrating that BHBA in noncytotoxic levels in our experiments suppressed LPS-induced inflammatory responses in microglia via attenuating expression of iNOS, COX-2, and proinflammatory cytokines.

### 3.4. Effects of BHBA Mediated by GPR109A

**Figure 5:**
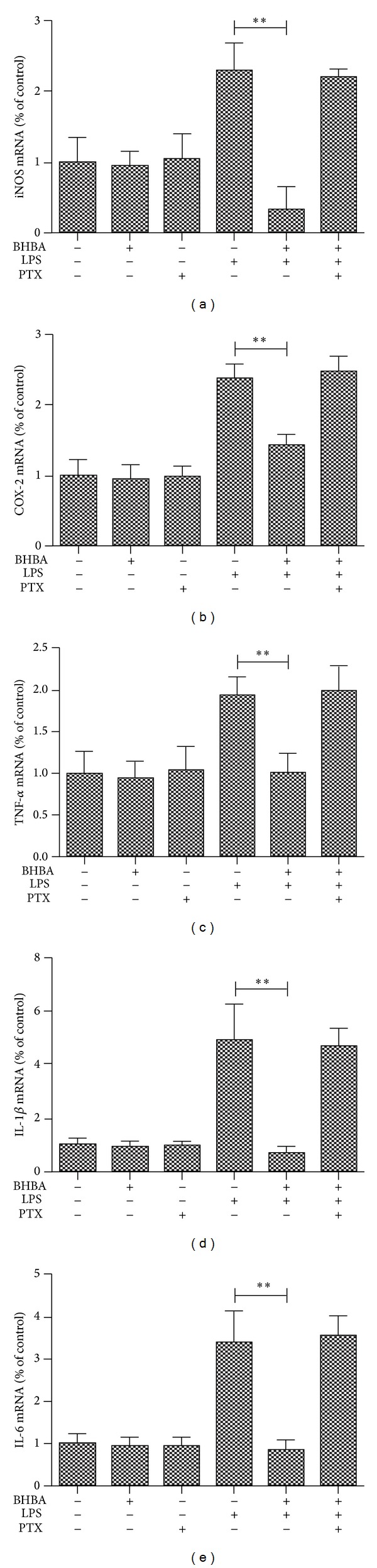
Effects of BHBA mediated by GPR109A. BV-2 cells were pretreated with vehicle or PTX for 1 h. Medium from each condition was removed and replaced with vehicle, BHBA (1.5 mM), PTX (100 ng/mL), LPS (1 *μ*g/mL), BHBA + LPS, or BHBA + LPS + PTX. BV-2 cells were sampled at 4 h. The mRNA of proinflammatory enzymes and proinflammatory cytokines were determined by quantitative real-time PCR. Attenuation by BHBA of induced mRNA of iNOS (a), COX-2 (b), TNF-*α* (c), IL-1*β* (d), and IL-6 (e) from BV-2 cells; this effect is abolished with pretreatment with PTX (**P < 0.01).

PTX is the ADP-ribosylating toxin produced by the whooping cough causing bacterium* Bordetella pertussis*. ADP-ribosylation of the *α* subunit of heterotrimeric G*α*i proteins locks the *α* subunits into an inactive state; thus it is unable to inhibit adenylyl cyclase [20]. It is widely applied as a tool in biochemical and pharmacological studies for the investigation of signaling pathways involving heterotrimeric G proteins [21]. In fact, PTX has been used as a tool in studies for action of GPR109A [22, 23]. As observed in previous experiments, preincubation with BHBA attenuated LPS-induced expression of iNOS (Figure 5(a)), COX-2 (Figure 5(b)), and TNF-*α* (Figure 5(c)), IL-1*β* (Figure 5(d)), and IL-6 (Figure 5(e)) mRNA in the cells of no pretreatment with PTX, but in the cells, which had been pretreated with PTX, this effect was abolished. In order to determine the cytotoxicity of PTX, we investigated the dose effect of PTX on cell viability by MTT assay. BV-2 cells were incubated with various doses of PTX for 24 h. The results of MTT assay showed that PTX, even at a high concentration of 1 *μ*g/mL, did not affect cell viability (data not shown).

### 3.5. BHBA Inhibits LPS-Induced NF-*κ*B Translocation and I*κ*B-*α* Degradation

**Figure 6:**
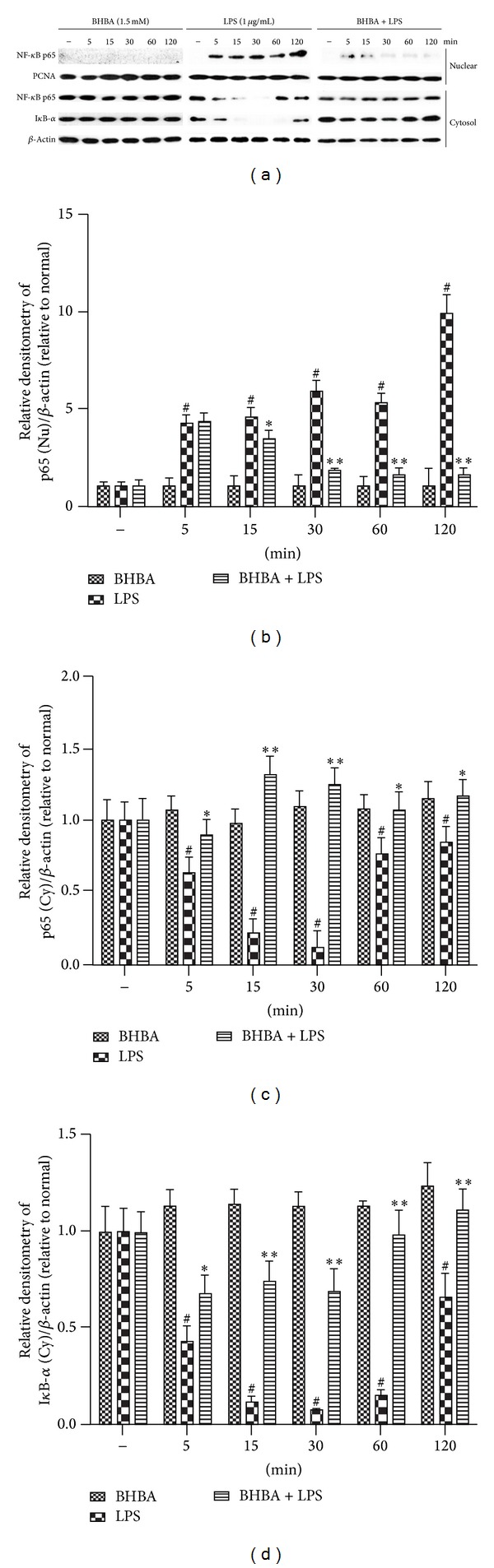
Effects of BHBA on NF-*κ*B translocation and I*κ*B-*α* degradation. BV-2 cells were pretreated with 1.5 mM BHBA 1 h prior to incubation of LPS (1 *μ*g/mL) for various time courses. NF-*κ*B p65 and I*κ*B-*α* in cytosol (Cy) and nuclear (Nu) fraction were determined by western blot. Each immunoreactive band was digitized and expressed as a ratio of *β*-actin or PCNA levels. The ratio of the control group band was set to 1.00. Data are expressed as mean ± SD of three independent experiments. ^#^Significant compared with LPS-untreated BHBA-stimulated group, *P* < 0.05. **P < 0.01, *P < 0.05, significantly different when compared with BHBA-untreated LPS-stimulated group.

The NF-*κ*B pathway is a key mediator of inflammation and is activated via Toll-like receptors (TLRs) resulting in increased cytokine and chemokine production [11]. Activation of NF-*κ*B and release of its subunits play a key role in the early development of neurodegenerative diseases [24]. Moreover, transcription of iNOS, COX-2, TNF-*α*, IL-1*β*, and IL-6 is regulated by the transcription factor NF-*κ*B. To further elucidate the mechanisms of BHBA on the inhibition of expression of iNOS, COX-2, and proinflammatory cytokines in BV-2 cells, the study examined the effect of BHBA on NF-*κ*B. BV-2 cells were pretreated with BHBA (1.5 mM) for 1 h and then stimulated with LPS (1 *μ*g/mL) for 5, 15, 30, 60, and 120 min. Nuclear and cytosolic extracts were isolated, and NF-*κ*B p65 subunits in the nuclear and cytosolic fractions were quantified by western blot. As shown in Figure 6, LPS sharply increased the translocation of NF-*κ*B p65 from cytosol to nucleus, and this increase was inhibited by pretreatment with BHBA (Figures 6(a)–6(c)). Because the LPS-mediated translocation of NF-*κ*B to nucleus is preceded by degradation of I*κ*B-*α*, we also examined protein levels of I*κ*B-*α* by western blot analysis. BHBA was found to inhibit the LPS-induced degradation of I*κ*B-*α* (Figures 6(a) and 6(d)). Moreover, only treatment with BHBA did not affect NF-*κ*B translocation and I*κ*B-*α* degradation. These results indicated that BHBA suppresses LPS-induced inflammatory responses, at least in part, through inhibiting LPS-induced NF-*κ*B translocation and I*κ*B-*α* degradation in BV-2 cells.

### 3.6. BHBA Does Not Suppress LPS-Induced Phosphorylation of MAPKs Family in BV-2 Cells

**Figure 7:**
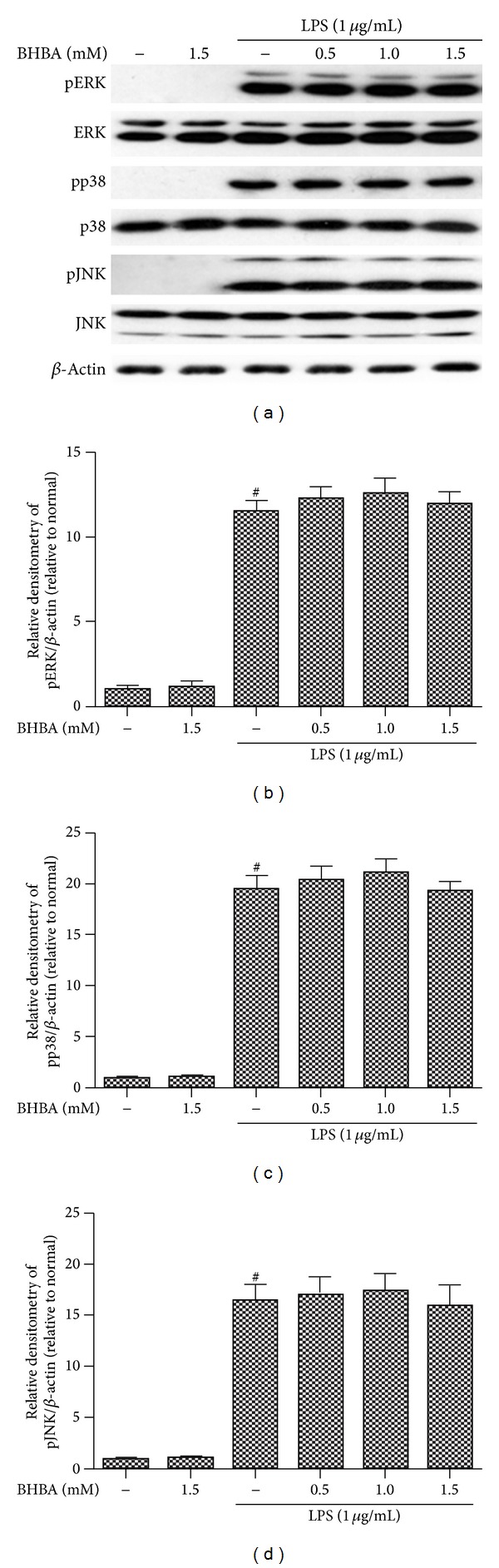
Effects of BHBA on LPS-induced phosphorylations of MAPKs. BV-2 cells were pretreated with or without BHBA (0.5, 1.0, and 1.5 mM) for 1 h and then were incubated with LPS (1 *μ*g/mL) for 30 min. Cell lysates were prepared and subjected to western blotting by using pJNK54/46, pp38, or pERK1/2 antibodies, respectively. Each immunoreactive band was digitized and expressed as a ratio of *β*-actin levels. The ratio of the control group band was set to 1.00. Data are expressed as mean ± SD of three independent experiments. ^#^Significantly different when compared with control alone, *P* < 0.05.

MAPK signaling pathways are known to play an important role in the regulation of inflammatory mediator production. Thus, we investigated the effects of BHBA on the activation of phosphor-ERK, phosphor-JNK, and phosphor-p38. According to our previous study upon LPS stimulation, phosphorylations of ERK, p38-MAPK, and JNK reached a maximum at 30 min and then decreased gradually in BV-2 cells. Then, we conducted treatments of LPS for 30 min combined with or without various doses of BHBA. Unexpectedly, as shown in Figure 7, in the presence of BHBA, the increased phosphorylations of ERK (Figures 7(a) and 7(b)), p38-MAPK (Figures 7(a) and 7(c)), and JNK (Figures 7(a) and 7(d)) upon LPS stimulation were not attenuated.

## 4. Discussion

Under conditions of inflammation associated with neurodegenerative diseases, microglial activation is believed to contribute to and/or exacerbate neuronal damage in neurodegenerative diseases [25, 26]. Since activated microglia produce neurotoxic factors such as ROS and proinflammatory cytokines, this could induce neuronal damage and/or neuronal death [17]. Additionally, microglia becomes highly reactive in response to neuronal damage and produces more neurotoxic factors [18]. Therefore, inhibition of microglial activation may be a potential therapeutic strategy to reduce neuronal cell death. This study shows, for the first time in murine microglial BV-2 cells, substantial anti-inflammatory effects of BHBA. BHBA significantly inhibits LPS-induced enhancement of expression of proinflammatory enzymes (iNOS and COX-2) and proinflammatory cytokines (TNF-*α*, IL-1*β*, and IL-6) in BV-2 cells, at both mRNA and protein levels, providing an underlying mechanism for the neuroprotective effects of BHBA in vitro.

COX-1 is constitutively expressed in most tissues, while COX-2 is induced during pathophysiological responses by inflammatory stimuli such as LPS, IL-1, and various growth factors in microglia and astrocytes [27, 28]. Reducing the activity of COX-2 can mitigate the progressive loss of dopaminergic neurons as well as the motor deficits caused by 1-methyl-4-phenyl-1,2,3,6-tetrahydropyridine (MPTP) neurotoxicity, possibly by suppressing the activation of microglia in the substantia nigra pars compacta (SNpc) [29]. In addition, the expression of iNOS and the overproduction of NO in microglia are considered to play a significant role in the pathogenesis of various neurodegenerative diseases. For instance, overproduction of NO by microglia contributes to the complication of AD and Parkinson's disease [30, 31]. Therefore, any substance that can attenuate expression of iNOS and COX-2 would be beneficial for delaying the progression of neurodegenerative disease. In the present study, BHBA (0.5, 1.0, and 1.5 mM) significantly inhibited the protein and mRNA expression of iNOS and COX-2 in a dose-dependent manner in LPS-stimulated BV-2 microglial cells, suggesting possible beneficial effects of BHBA by attenuating the activation of microglial cells and subsequent production of inflammatory mediators.

Microglia activation, in turn, causes release of proinflammatory cytokines, including TNF-*α*, IL-1*β*, and IL-6 [32]. In normal circumstances, such response by microglia is protective in fighting off pathogens like bacteria, for example. In contrast, under pathological conditions induced by certain insults, including oxidative stress, excitotoxicity, microglia can be overstimulated and produce excess proinflammatory cytokines that exacerbate neuronal damage in neurodegenerative diseases [33]. This study investigated whether BHBA inhibits LPS-induced production of proinflammatory cytokines in BV-2 cells. Our data suggest that BHBA significantly reduces LPS-induced protein and mRNA expression levels of TNF-*α*, IL-1*β*, and IL-6. These results suggest that BHBA has the potential to act directly on microglia to inhibit proinflammatory cytokines that may contribute to its neuroprotective effects in vivo.

To further characterize the nature of the inhibitory effect of BHBA on proinflammatory proteins production, the NF-*κ*B signal transduction pathway, which was activated by LPS in microglia, was examined. NF-*κ*B is clearly one of the most important regulators of proinflammatory gene expression. Synthesis of cytokines, such as TNF-*α*, IL-1*β*, and IL-6, is mediated by NF-*κ*B, as is the expression of COX-2 and iNOS [11]. In resting cells, NF-*κ*B is retained in the cytoplasm by binding to I*κ*B-*α*. Activation of NF-*κ*B occurs via phosphorylation of its endogenous inhibitor I*κ*B-*α* that results in the release and nuclear translocation of active NF-*κ*B [34–36]. The results suggest that incubation of BV-2 cells with LPS cause a marked degradation of cytosolic I*κ*B-*α* and NF-*κ*B p65 translocation into the nucleus, but pretreatment with BHBA significantly inhibited both of the I*κ*B-*α* degradation and NF-*κ*B p65 nuclear translocation. These results indicate that BHBA suppresses LPS-induced inflammatory responses, at least in part, through inhibiting LPS-induced NF-*κ*B translocation and I*κ*B-*α* degradation in BV-2 cells. Furthermore, we demonstrated that the inhibitory effect of BHBA is mediated by GPR109A. However, how GPR109A regulates NF-*κ*B activation in microglia remains unknown.

The role of cyclic adenosine monophosphate (cAMP) in this process remains to need further research, as previous studies have given controversial results. It has been shown that BHBA decreases lipolysis by inhibition of cAMP production through G_*α*_i-mediated secretion of adiponectin in adipose tissue [23]. On the other hand, cAMP levels are also a key element for activation of immune cells [37]. To date, most studies on the effects of cAMP on microglia function have focused on its anti-inflammatory effects on fully activated microglia. For example, in LPS-activated microglia, agents that increase cAMP levels decrease inflammatory cytokine release, inhibit phagocytosis, and reduce reactive oxygen [38–41]. From the present results, it appears that cAMP can also act as a positive regulator of a number of immune function genes, including some normally considered to be proinflammatory. Agarwal et al. have found that increased bacteria-derived cAMP within macrophages results in cAMP response element-binding protein (CREB) phosphorylation and TNF-*α* production [42]. It has been shown in several systems that LPS can increase cAMP in microglia [13], and in this regard a subset of cAMP-induced gene transcription could be viewed as part of a normal inflammatory response. Thus, further investigation is necessary to determine whether the mechanism of reduction of NF-*κ*B translocation and I*κ*B-*α* degradation by BHBA is cAMP dependent.

MAPKs family has been shown to play important roles in LPS- induced iNOS, COX-2, and proinflammatory cytokines expression in BV-2 cells [43]. Therefore, we investigated the effect of BHBA on phosphorylation of three MAPKs induced by LPS in BV-2 cells. Unexpectedly, in the presence of BHBA, the increased phosphorylations of JNK, ERK, and p38-MAPK upon LPS stimulation were not attenuated. These results suggest that BHBA- mediated attenuation of proinflammatory mediators is not associated with downregulation of the MAPK signaling pathway.

In summary, the results of this study provide evidence that BHBA might exhibit its anti-inflammatory effects via activating GPR109A and suppressing NF-*κ*B translocation and I*κ*B-*α* degradation. This finding provides a new molecular insight into the mechanism by which BHBA exerts its anti-inflammatory function. Arising from the above, we suggest that BHBA might be a strong candidate for treatment of neurodegenerative diseases.

## Figures and Tables

**Table 1:** The primer sequences of *β*-actin, GPR109A, iNOS, COX-2, TNF-*α*, IL-1*β*, and IL-6.

Gene	Sequences	Length (bp)
*β*-Actin	(F) 5′-GTCAGGTCATCACTATCGGCAAT-3′	147
	(R) 5′-AGAGGTCTTTACGGATGTCAACGT-3′	
GPR109A	(F) 5′-GCTGCCCTGTCGGTTCAT-3′	134
	(R) 5′-CGTGGCTGACTTTCTCCTGAT-3′	
iNOS	(F) 5′-GAACTGTAGCACAGCACAGGAAAT-3′	158
	(R) 5′-CGTACCGGATGAGCTGTGAAT-3′	
COX-2	(F) 5′-CAGTTTATGTTGTCTGTCCAGAGTTTC-3′	127
	(R) 5′-CCAGCACTTCACCCATCAGTT-3′	
TNF-*α*	(F) 5′-GCAACTGCTGCACGAAATC-3′	136
	(R) 5′-CTGCTTGTCCTCTGCCCAC-3′	
IL-1*β*	(F) 5′-GTTCCCATTAGACAACTGCACTACAG-3′	139
	(R) 5′-GTCGTTGCTTGGTTCTCCTTGTA-3′	
IL-6	(F) 5′-CCAGAAACCGCTATGAAGTTCC-3′	138
	(R) 5′-GTTGGGAGTGGTATCCTCTGTGA-3′	
